# Prognostic value of MTV and TLG of 18 F-FDG PET in patients with head and neck squamous cell carcinoma: A meta-analysis

**DOI:** 10.1097/MD.0000000000030798

**Published:** 2022-09-30

**Authors:** Aihua Jin, Xing Lin, Xuezhe Yin, Yinfeng Cui, Liguang Ma

**Affiliations:** a Department of Clinical Laboratory, Yanbian University Hospital, Yanji, Jilin Province, China; b Department of Thoracic Surgery, Yanbian University Hospital, Yanji, Jilin Province, China; c Department of Respiration Medicine, Yanbian University Hospital, Yanji, Jilin Province, China; d Department of Stomatology, Medical College of Yanbian University, Jilin Province, China.

**Keywords:** head and neck squamous cell carcinoma, meta-analysis, MTV, PET/CT, TLG

## Abstract

The current systematic review and meta-analysis explored the value of metabolic tumor volume (MTV) as well as total lesion glycolysis (TLG) in predicting the prognosis of head and neck squamous cell carcinoma (HNSCC) using 18 F-FDG PET parameters.

**Methods:**

This work identified relevant studies in the English language by searching several electronic databases, like Cochrane Library, EMBASE, and PubMed. In addition, pooled hazard ratios (HRs) were also calculated to analyze whether MTV and TLG were significant in predicting prognosis.

**Results:**

The present study included 15 primary studies involving HNSCC cases. As for the elevated TLG, it attained the pooled HR of 1.85 (95% confidence interval [CI], 1.16–2.94; *P* = .000; *I*^2^ = 78.3%) in predicting overall survival (OS), whereas that for elevated MTV was1.22 (95%CI, 1.09–1.36; *P* = .000; *I*^2^ = 82.4%). Besides, for elevated MTV, it attained the pooled HR of 1.34 (95%CI, 1.15–1.56, *P* = .000; *I*^2^ = 86.0%) in predicting disease-free survival (DFS); while the elevated TLG was related to DFS. Sensitivity analysis confirmed that our results are reliable. As for MTV, the ROC-stratified subgroups for DFS and multivariate analyses-stratified subgroups for OS showed statistically significant differences, with no obvious heterogeneities across different studies. For TLG, other methods-stratified subgroups for OS showed statistically significant differences, with no obvious heterogeneity across different studies.

**Conclusion:**

This work indicated that PET/CT is of predictive significance across HNSCC cases. Although the included articles used different methods and recruited HNSCC cases with high clinical heterogeneity; however, our findings confirmed that an elevated MTV can predict the increased risk of side reactions or even death among HNSCC cases and that an elevated TLG can predict a higher death risk.

## 1. Introduction

Head and neck cancer is the 6^th^ leading cancer globally by morbidity rate and accounts for about 800,000 newly diagnosed cancer cases annually.^[1]^ Head and neck squamous cell carcinoma (HNSCC) show high heterogeneity and can involve several subsites, like salivary glands, oropharynx, nasopharynx, larynx, and hypopharynx. The HNSCC is usually diagnosed at the advanced stage.^[2]^ Also, many patients with HNSCC have disease progression in 3 to 5years,^[3]^ with the risks of local and distant metastases being 60% and 30%, respectively.^[4]^ Although great progress has been made in HNSCC related treatment and research over the past few decades; however, patient survival is not greatly improved, with the 5-year overall survival (OS) being <50%.^[5]^ Classical prognostic factors alone cannot accurately predict clinical outcomes in patients with HNSCC as the tumor behaviors and molecular mechanisms underlying HNSCC are heterogeneous. Therefore, great attention is being paid to the identification of early diagnostic, prognostic biomarkers, and therapeutic targets.

Notably, 18-fluorodeoxyglucose (18F-FDG) positron emission tomography (PET/CT) is recognized as an effective tool in predicting and assessing cancer as well as in determining its TNM (tumors, nodes, and metastases) stage. The FDG parameters, like tumor volume/metabolism, total lesion glycolysis (TLG), maximal standard uptake value, and metabolic tumor volume (MTV), have been widely investigated. TLG refers to the product of mean SUV and MTV, while MTV stands for the size of tumor tissue ingesting18 F-FDG actively.^[6–9]^ However, it remains controversial to predict HNSCC prognosis based on 18F-FDG PET/CT parameters. In some articles, the higher MTV predicted poor prognosis for HNSCC patients,^[10,11]^ whereas the findings by Hidenori et al^[12]^ do not support this relation. Consequently, this present meta-analysis is focused on examining whether TLG and MTV have some significant benefits in the prognosis of HNSCC.

## 2. Material and methods

### 2.1 Registration

The current systematic review and meta-analysis were performed in adherence to the preferred reporting items of the systematic review and meta-analysis guidelines.^[13]^ The present study utilized data obtained from previously published articles, so ethical approval and patient consent were waived off.

### 2.2 Search strategy and study selection criteria

This work involved the identification of relevant articles within Cochrane Library, EMBASE, and Pubmed databases between February 2012and May 2021 using the following search terms: “head and neck” OR “oropharynx” OR “oral cavity” OR “larynx” OR “hypopharynx” AND “carcinoma” OR “carcinoma” AND “positron emission tomography” OR “positron emission tomography-computed tomography” OR “PET-CT” OR “PET” OR “PET/CT” OR “PET CT” OR “FDG” OR “fluorodeoxyglucose” AND “prognostic” OR “outcome” OR “prognosis” OR “predictive” OR “survival.” The study inclusion criteria included patients with histologically confirmed HNSCC; studies documenting 18F-FDG PET/CT as the imaging approach before treatment; studies reporting at least 1 type of survival data; and articles published in the English language. The study exclusion criteria were as follows: studies regarding disease diagnosis and stage classification, with disease progression or recurrence; studies that involved disease recurrence before treatment; and reviews, case reports, conference abstracts, or editorial materials. Two reviewers were responsible for the study retrieval and selection in line with those pre-determined standards. Any disputes were resolved through mutual negotiation.

### 2.3 Data collection

The data from all the relevant studies were collected independently by 2 reviewers, which included basic information about the included studies, like the first author, publication year, study design, study implementation time, as well as follow-up length; baseline patient demographics and tumor characteristic, including the number of cases, median age, histology, TNM classification, therapeutic measures, and endpoints. Besides, this work extracted data such as fasting time before injection, truncated interval for FDG infection dose, 18F-FDG PET data, as well as truncated values for PET parameters, like tumor profiles, MTV, and TLG.

### 2.4 Statistical analysis

The statistical analysis for this study was conducted using the same method as that used previously.^[14]^ OS indicated the period from treatment initiation to the date of mortality from any cause. The present work pooled recurrence-free survival (RFS), disease-free survival (DFS), progression-free survival (PFS), laryngectomy-free survival (LFS), event-free survival (EFS), and DFS from all those enrolled articles and redefined DFS.^[15]^ In addition, hazard ratios (HRs) along with relevant 95% confidence intervals (CIs) were pooled, and 18 F-FDG PET parameters’ impaction disease prognosis according to HR effect size were analyzed to explore the relation of TLG/MTV with HNSCC prognosis. HR < 1 indicated survival benefit for patients who had higher TLG or TMV, whereas HR > 1 stood for poor prognosis. Chi-square Q test along with *I*^2^ statistics was utilized to measure statistical heterogeneity. *P* < .05 was set as the significant heterogeneity level, and a random-effects model was employed; while, *I*^2^ > 50% stood for the absence of heterogeneity, and so a fixed-effects model was utilized. All statistical analyses were performed using RevMan version 5.3 (The Nordic Cochrane Centre, The Cochrane Collaboration) and STATA (version 12.0; STATA Corp., College Station, TX). Begg’s and Egger’s tests were applied for publication bias assessment while using STATA version 12.0. *P* < .05 stood for statistical significance. An analysis by trim and fill method was carried out when Egger’s and Begg’s tests indicated possible publication bias to ensure that our pooled HRs were reliable.

## 3. Results

### 3.1 Study screening results

**Figure 1.**
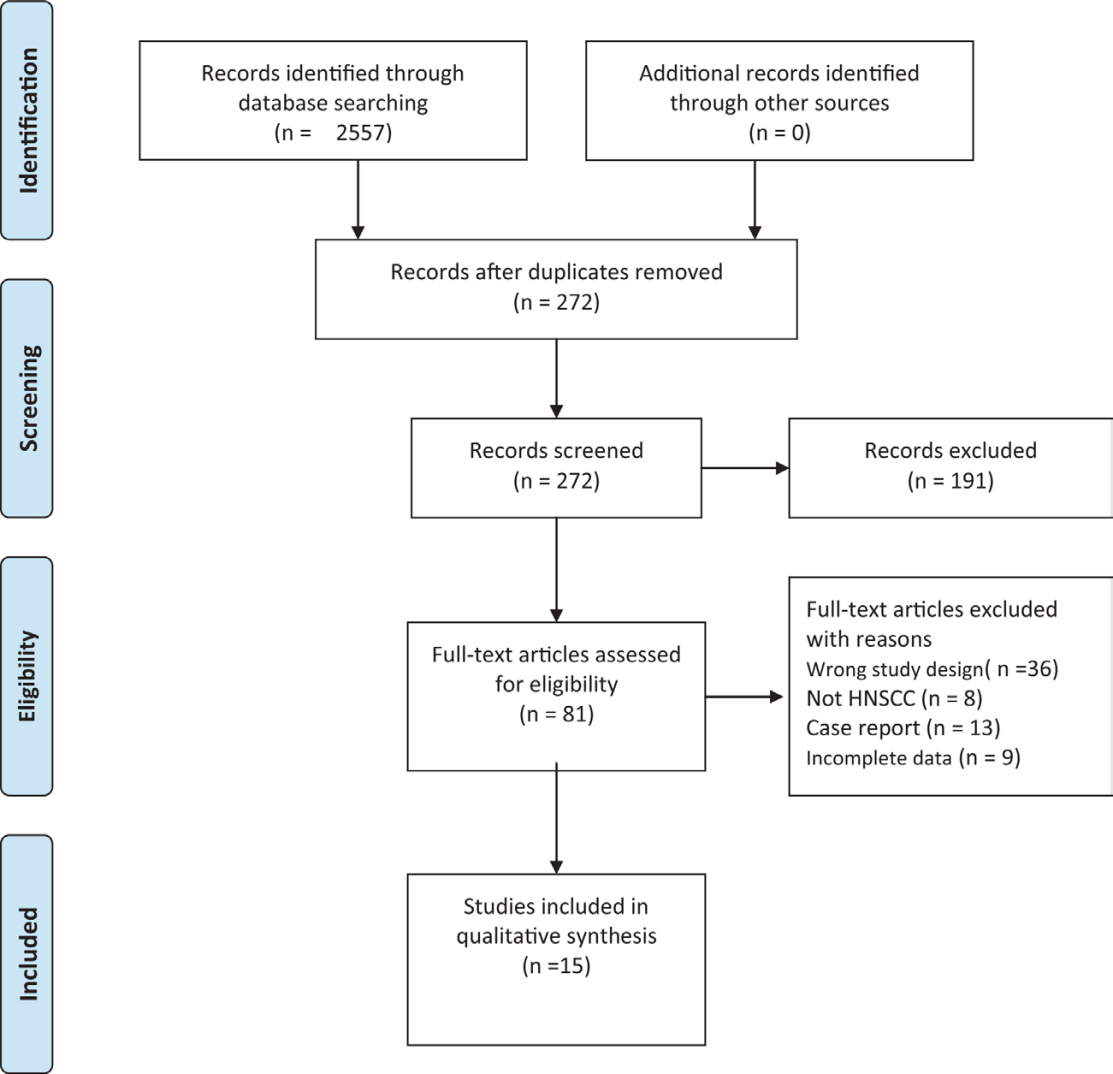
Flowchart of our study screening process.

Figure 1 presents the study screening flowchart across 3 databases. Of 2557 articles identified for the study, 1520 were from PubMed, 1037 from EMBASE, and none from Cochrane Library. A total of 15 studies recruiting 1184cases were ultimately included in this analysis according to our pre-determined criteria. These included studies were published during 2012 to 2021^[10–12,16–27]^ (Fig. 1) and documented the significance of MTV or TLG in predicting prognostic outcomes in patients with HNSCC.

### 3.2 Study features

Of 15 studies included, 8 studies were conducted within Asian countries, including China (2), Japan (3), and South Korea (3), while the remaining were conducted in the USA (3), Canada (1), Finland (1), Netherlands (1) and Spain (1). The studies included were published during 2012–2021, had a sample size of 22 to 168, and included 13 retrospective and 2 prospective articles. Of the articles that were included, 3 articles had PFS, 5 had DFS, 2each had RFS and LFS, 1 had EFS documented, while13 reported OS. The follow-up period in those selected articles ranged between 3 to 101 months. These 15 articles also analyzed one or more histological features along with the rapeutic strategies. Table 1 displays study characteristics, histology, and therapeutic strategy. Additionally, the FDG dose in these studies ranged from 200MBq to 490MBq. Table 2 enlists the fasting period post-injection interval with the determination of thresholds.

**Table 1:** Enrolled study characteristics.

Study	Yr	Country	Study period	Follow-up duration (mo)	Median age (range), yr	No. of patients	TNM staging	End points	Study design	Histology	Treatment
Hidenori Suzuki et al	2021	Japan	2004- 2016	4.65 ± 2.87 yrs	68	46	III-IV	DFS,OS	R	Locally advanced LaryngealSCC (LALSCC)	RT CRT
Hyukjin Yoon et al	2021	Korea	2007 -2017	62	61 (35–91)	119	II-IV	DFS,OS	R	locally advanced HNSCC	RT
Jefferson Rijo-Cedeño et al	2021	Spain	2012 -2018	60	65 (38–87)	62	III/IV	DFS,OS	R	HNSCC	RT chemotherapy
Roland M. Martens et al	2020	Netherlands	2014-2018	-	64(57.8–69.3)	70	II-IV	RFS,OS	P	HNSCC	RT chemotherapy
JunjiMiyabe et al	2017	Japan	2007 - 2015	45(12–101)	66(43–79)	101	III/IV	LFS,OS	R	laryngeal or hypopharyngeal squamous cell carcinoma	RT chemotherapy
Ying-Chun Lin et al	2016	China	2007-2013	24 (6 to 72)	53 (32 -78)	76	I-IV	RFS	R	oropharyngeal and hypopharyngeal SCC	CRT or RT
Han Zhang et al	2016	Canada	2008- 2012.	1.4(1.3–5.2) years	61.7 (23–88)	122	I-IV	DFS,OS	R	Oral cavity squamous cell carcinoma (OCSCC)	Surgery CRT or RT
Hiroshi Hoshikawa et al	2015	Japan	2006- 2013.	56 (, 14–96)	66 (40–83)	53	II-IV	LFS,OS	P	HNSCC	CRT
Ying-Chun Lin et al	2015	China	2007 -2012	18 (3–69)	52 (37–78)	91	III/IV	DFS,OS	R	pharyngeal cancers with histologic proof of squamous cell carcinoma	RT CRT
Krishna C. Alluri et al	2014	USA	2004-2011	31(3–97)	58.8 (29–78)	70	III/IV	EFS	R	Oropharyngeal Squamous Cell Carcinoma	surgery CRT RT
Gaber Komar et al	2014	Finland	2005- 2007	41 ± 15	56 ± 12	22	I-IV	OS	R	HNSCC	CRT surgery
VasaviPaidpally et al	2014	USA	2004 - 2012	17.5 (8.0–37.5).	57 ± 14	34	I-IV	OS	R	head and neck squamous cell can- cer (HNSCC)	CRT
Chad Tang et al	2012	USA	2003-2009.	24 (1.4–85)	58 (14–89)	168	I-IV	PFS,OS	R	head and neck squamous cell can- cer (HNSCC)	RT
G. C. Park et al	2012	Korea.	2004-2009.	40.4 (24.5–90.1	65(34–81)	81	III/IV	OS	R	advanced-stage squamouscell carcinoma of the laryngohypopharynx	Surgery RT CRT chemotherapy
Seung Hwan Moon et al	2012	Korea	2004 to June 2010	25.7 (7.6–77.1)	56.5 +- 9.7	69	I-IV	OS	R	Squamous cell carcinoma Squamous cell carcinoma variant	RT Surgery CRT chemotherapy

CRT = chemoradiotherapy, DFS = disease-free survival, LFS = laryngectomy-free survival, OS = overall survival, P = prospective,PFS = progression-free survival, R = retrospective, RFS = recurrence-free survival, RT = radiotherapy.

**Table 2.** 18 F-FDG PET imaging methods for enrolled articles.

Study	Duration of fasting	Post-injection interval	Dose of 18 F-FDG	Determination of cut- off values	Cutoff values	
					MTV (cm^3^)	TLG
Hidenori Suzuki et al	-	-	-	Others	13.1	46.5
Hyukjin Yoon et al	-	-	-	Others	-	-
Jefferson Rijo-Cedeño et al	6h	50–60 min	350–400 MBq	ROC	37	247
Roland M. Martens et al	-	60 min	2.5 MBq/kg	Others	-	-
JunjiMiyabe et al	4 h	60 min	3.7 MBq/kg	ROC	28.7	-
Ying-Chun Lin et al	4 h	60 min	370 MBq	ROC	14.5	-
Han Zhang et al	4 h	60-min	5.18 MBq/kg	Others	-	-
Hiroshi Hoshikawa et al	-	-	-	ROC	8.9	91.9
Ying-Chun Lin et al	4 h	60 min	370 MBq	Others	14.5	-
Krishna C. Alluri et al	-			Others	-	-
Gaber Komar et al	-	60 min	200–389 MBq	Others	-	-
VasaviPaidpally et al	-	-		ROC	-	-
Chad Tang et al	8 h	45-60 min		Others	-	-
G. C. Park et al	6 h	-	490 MBq	Others	18	-
Seung Hwan Moon et al	6 h	-	5.5 MBq/kg	ROC	-	-

MTV = metabolic tumor volume, ROC = receiver operating characteristic, TLG = total lesion glycolysis.

### 3.3 Quality assessment of enrolled articles

**Figure 2.**
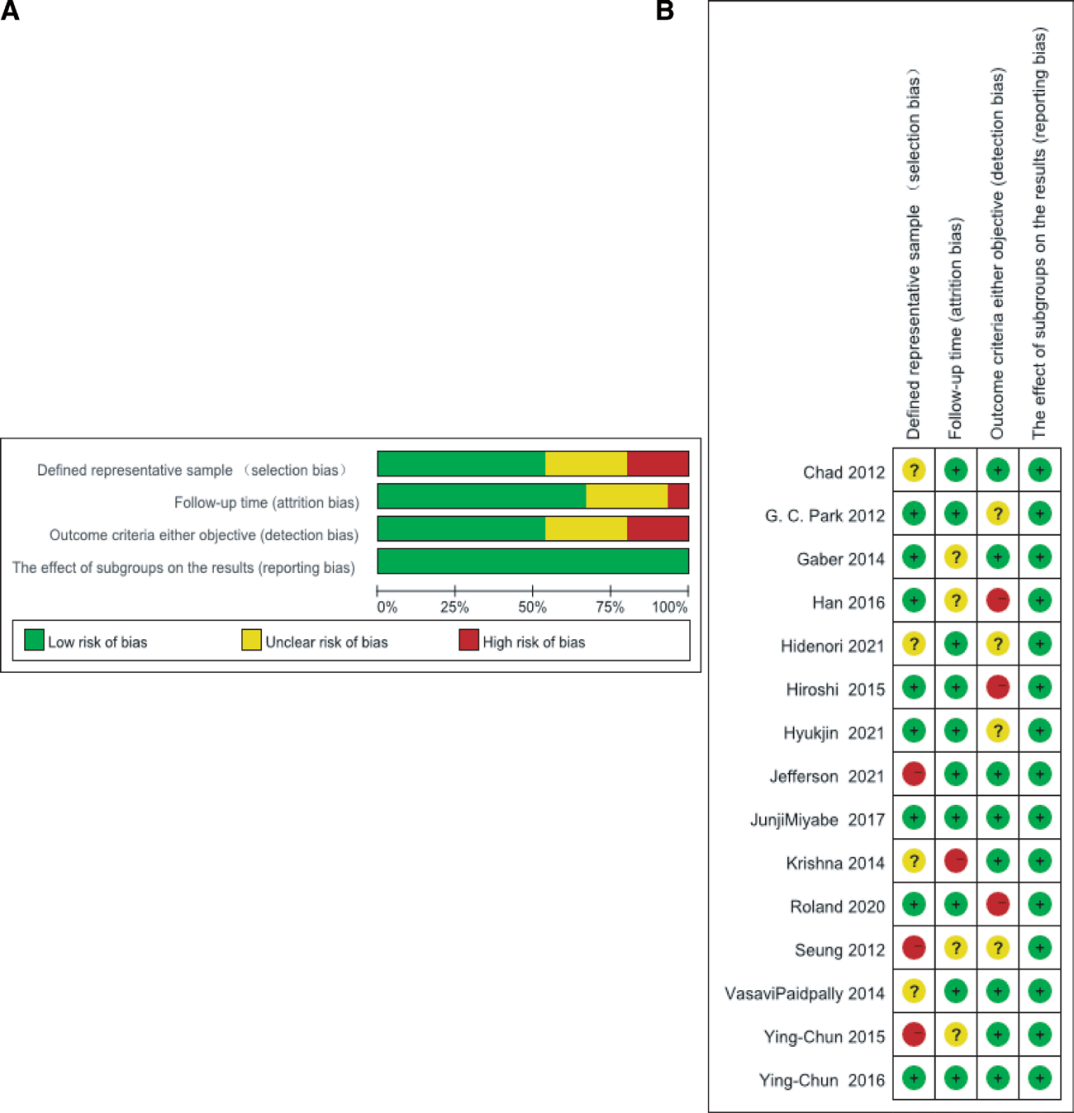
(A) Diagram illustrating the bias risk: Judgment by researchers with regards to bias risk items displayed as percentages in those included studies. (B) Summarization of the bias risk: Judgment by researchers with regards to the bias risk items in those included studies.

Study quality was assessed using guidelines of the Critical Appraisal of Prognostic Studies (https://www.researchgate.net/publication/292612152_Critical_Appraisal_of_Prognostic_Studies) (Fig. 2). As a result, all the enrolled articles were of high quality; however, 7 studies still had a high or unclear bias risk because of the small sample size. Also, 7 studies had a high or unclear bias risk in objective measurement or outcome criteria as some data were missing, while 5 studies had a higher bias risk in follow-up length measurement due to the short follow-up length or the missing related data. Many of these studies were well described and reported adverse reactions by objective standards.

### 3.4 Primary endpoint: DFS

**Figure 3.**
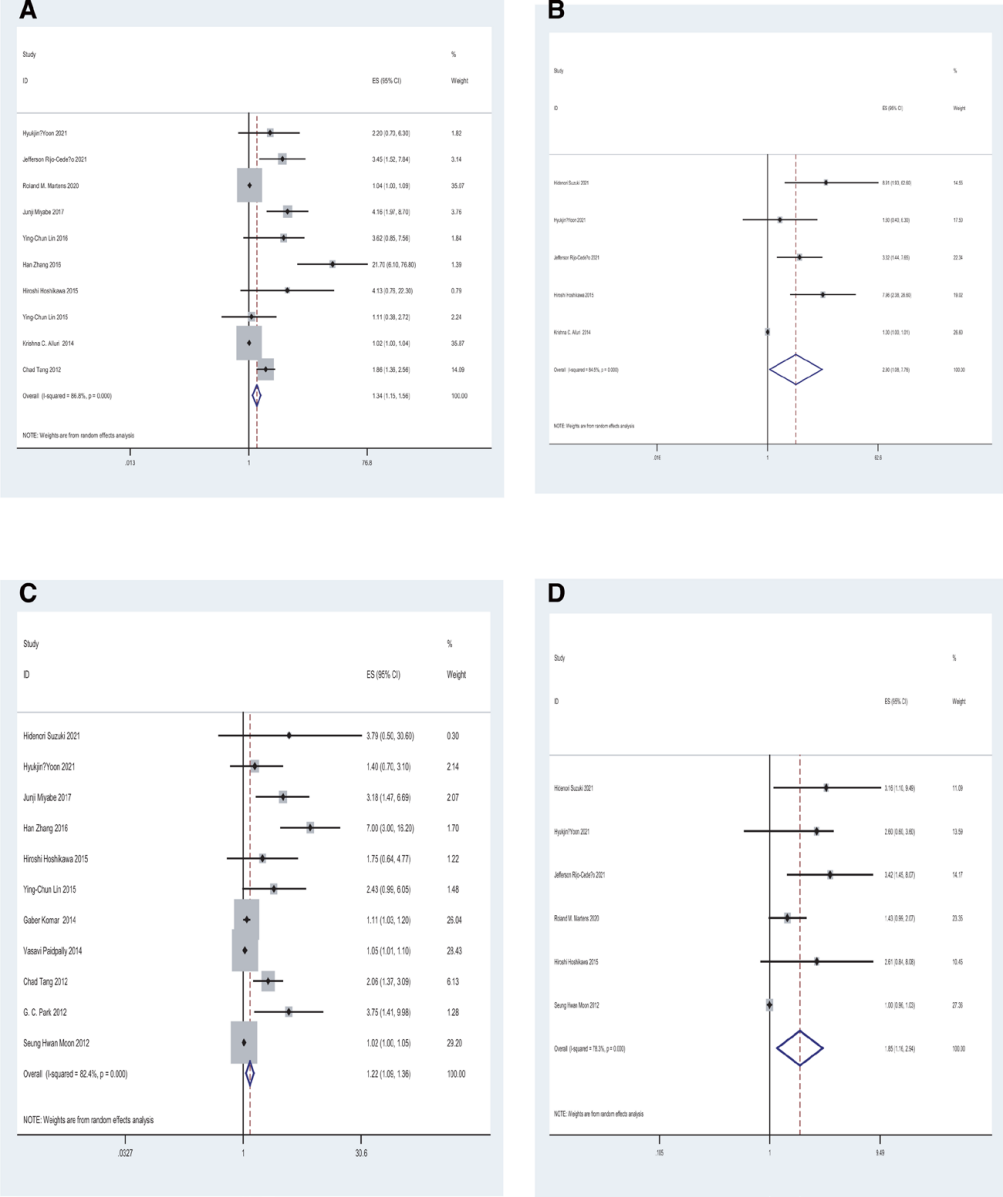
Forest plots showing the HRs of DFS with MTV (A) and TLG (B), as well as OS with MTV (C) and TLG (D). The heterogeneity was measured by the chi-square test. *P* < .05 stands for distinct heterogeneity. Horizontal lines = 95% CIs. Squares = point estimate of single articles. Rhombus = summarized estimate together with relevant 95%CI. DFS = disease-free survival, fixed, fixed-effects model, HRs = hazard ratios, MTV = metabolic tumor volume, random, random-effects model, OS = overall survival, TLG = total lesion glycolysis.

**Figure 4.**
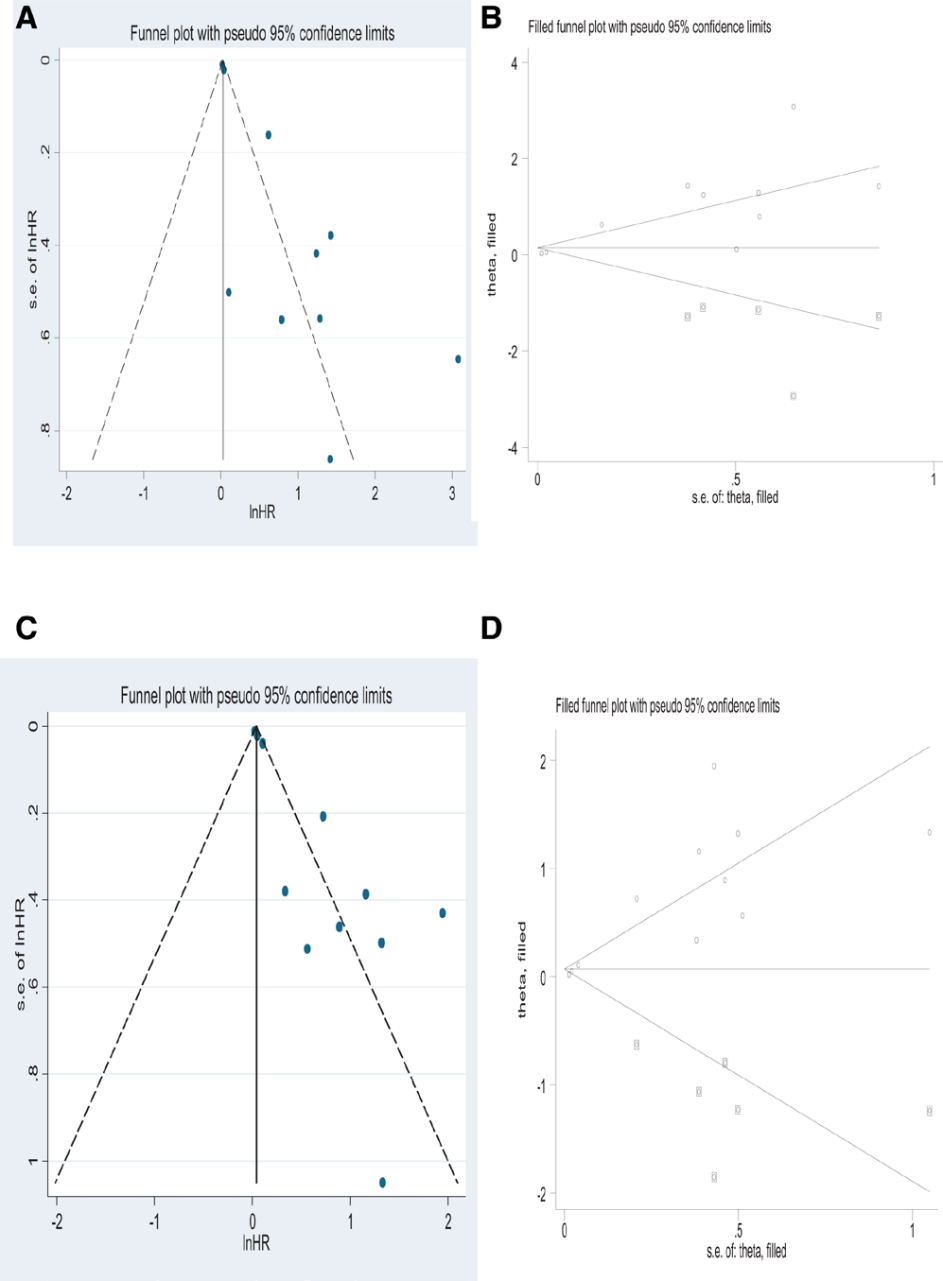
Funnel plots showing DFS with (A) and without (B) using trim and fill method; Funnel plots for OS with (C) and without (D) using trim and fill method. Funnel plots were drawn after calculating pseudo-95% CI WAs corresponding to the relevant 95% CI of specific standard error. DFS = disease-free survival, HR, hazard ratio, OS = overall survival.

A total of 10 studies analyzed MTV and DFS. The pooled HRs suggested that a higher MTV was indicative of dismal DFS. There was a statistical difference observed among studies upon applying the fixed effects model (HR = 1.03; 95% CI = 1.01–1.06, *P* = .000; *I*^2^ = 86.0%), showing the presence of heterogeneity across diverse works, and significant observations were obtained from the random-effects model (HR = 1.34; 95% CI = 1.15–1.56, [Fig. 3A]). In the current work, sensitivity analysis was done to estimate the impact on HRs, and so, the elimination of any individual work did not markedly affect the overall results (Fig. S1A, http://links.lww.com/MD/H397), indicative of the reliability of results. According to Funnel plots, there was distinct publication bias observed (Fig. 4A), and so, we carried out Begg’s and Egger’s tests for evaluating the potential publication bias. Begg’s test (*P* = .474) revealed no obvious publication bias; however, Egger’s test (*P* = .001) suggested a possible publication bias (Fig. S2A, http://links.lww.com/MD/H398). Consequently, the trim and fill method was used to ensure the reliability of combined HRs. There after, the symmetry of funnel plots was observed with no distinct alteration in results before and after the addition of hypothesis literature (HR = 1.027; 95% CI = 1.008–1.045; [Fig. 4B]). This indicated that DFS and MTV were markedly related. Subgroup analyses were also performed after stratification by analysis, cutoff method, region, and endpoint (Table 3). According to the subgroup analysis stratified by region, the 5 studies conducted in Asia (HR = 2.75;95% CI = 1.75–4.33; *P* = .279) and 3 studies conducted in America (HR = 2.41; 95% CI = 1.07–5.43) exhibited a significant correlation. However, 2 studies conducted in Europe did not exhibit any obvious correlation (HR = 1.76; 95% CI = 0.55–5.65). The HR of 4 articles using ROC-based threshold was 3.81 (95%CI = 2.37–6.10), while that of6 articles using other methods was 1.15 (95%CI = 1.01–1.30; *P* = .000). According to the subgroup analysis stratified by the analysis method, the HR of 5 studies employing multivariate regression analysis was 3.86 (95%CI = 1.30–11.47, *P* = .000) and showed a significant association, whereas that of 5 studies employing univariate regression analysis exhibited no association (HR = 1.57; 95% CI = 0.98–2.51). In terms of endpoint, we categorized relevant studies into DFS, RFS, LFS, EFS, and PFS groups, respectively. DFS group (HR = 3.50; 95% CI = 1.17–10.48), LFS group (HR = 4.16; 95% CI = 2.11–8.20), PFS group (HR = 1.86; 95% CI = 1.36–2.55) showed correlations; while RFS group (HR = 1.71; 95% CI = 0.52–5.68) and EFS group (HR = 1.02; 95% CI = 1.00–1.04) showed no correlations.

**Table 3:** Subgroup of DFS with MTV and OS with MTV and TLG.

Volumetric parameters	Factor	No. of studies	Heterogeneity test (*I*^2^, *P*)	Effect model	HR	95%CI of HR	Conclusion
MTV	region						
	Asian	5	21.3, .279	fixed	2.75	1.75,4.33	significant
	Europen	2	87.8, .004	random	1.76	0.55,5.65	insignificant
	American	3	94.5, .000	random	2.41	1.07,5.43	significant
	Cutoff method						
	ROC	4	0.0, .988	fixed	3.81	2.37,6.10	significant
	Others	6	87.0, .000	random	1.15	1.01,1.30	significant
	Analysis method						
	Multivariate analysis	5	91.3, .000	random	3.86	1.30,11.47	significant
	Univariate analysis	5	80.8, .000	random	1.57	0.98,2.51	insignificant
	Endpoint						
	DFS	4	78.1, .003	random	3.50	1.17,10.48	significant
	RFS	2	80.0, .026	random	1.71	0.52,5.68	insignificant
	LFS	2	0.0, .994	fixed	4.16	2.11,8.20	significant
	EFS	1	-	-	1.02	1.00,1.04	insignificant
	PFS	1	-	-	1.86	1.36,2.55	significant
MTV	region						
	Asian	7	73.0, .001	random	1.96	1.16,3.31	significant
	American	3	93.3, .000	random	2.23	0.96,5.19	insignificant
	European	1	-	-	1.11	1.03,1.20	significant
	Cutoff method						
	ROC	4	72.0, .013	random	1.05	0.98,1.13	insignificant
	Others	7	83.3, .000	random	2.29	1.35,3.91	significant
	Analysis method						
	Multivariate analysis	4	0.0, .576	fixed	4.29	2.67,6.89	significant
	Univariate analysis	7	70.8, .002	random	1.09	1.01,1.17	significant
TLG	region						
	Asian	4	74.1, .009	random	1.91	0.91，3.99	insignificant
	European	2	70.0, .068	random	2.02	0.88，4.66	insignificant
	Cutoff method						
	ROC	3	81.3, .005	random	1.88	0.74，4.79	insignificant
	Others	3	32.0, .230	fixed	1.67	1.20，2.31	significant
	Analysis method						
	Multivariate analysis	5	80.3, .000	random	1.77	1.09,2.88	significant
	Univariate analysis	1	-	-	2.61	0.84,8.09	insignificant

CI = confidence interval, DFS = disease-free survival, HR = hazard ratio, LFS = laryngectomy-free survival, MTV = metabolic tumor volume, PFS = progression-free survival, RFS = recurrence-free survival, ROC = receiver operating characteristic, TLG = total lesion glycolysis.

A total of 5 studies reported DFS and TLG. After combing HRs, the random-effects model was applied for detecting statistical significance (HR = 1.34; 95% CI = 1.15–1.56; *P* = .350; *I*^2^ = 4.7%) (Fig. 3B). The present study conducted a sensitivity analysis to assess the impact of combined HRs (Fig. S1B, http://links.lww.com/MD/H397). As a result, when studies by Hidenori et al,^[12]^ Jefferson et al,^[20]^ and Hiroshi et al^[19]^ were eliminated in succession, no significant correlation was detected.

### 3.5 Secondary endpoint: OS

A total of 11 studies analyzed OS with MTV. After combining HRs, the random-effects model was used for detecting statistical significance (HR = 1.22; 95% CI = 1.09–1.36, *P* = .000; *I^2^* = 82.4%; Fig. 3C). Based on the sensitivity analysis performed to analyze the impact on combined HRs, no distinct alterations were observed after all studies were removed successively (Fig. S1C, http://links.lww.com/MD/H397), indicative of the reliability of results. According to Funnel plots, there was a distinct publication bias observed (Fig. 4C), and so, Begg’s and Egger’s tests were performedfor evaluating potential publication bias. Begg’s test (*P* = .350) showed no significant publication bias, whereas Egger’s test (*P* = .000) suggested possible publication bias (Fig. S2B, http://links.lww.com/MD/H398). Consequently, the trim and fill method of analysis was used to ensure the reliability of combined HRs. The symmetry of funnel plots was obtained subsequently, with no distinct alterations of results before and after adding hypothesis literature (HR = 1.036; 95% CI 1.014–1.058; [Fig. 4D]). This indicated that MTV and OS were markedly related. In addition, a subgroup analysis was also performed upon stratification by analysis, cutoff method, and region (Table 3). According to subgroup analysis stratified by region, the HR of 7 Asian studies (HR = 1.96;95% CI = 1.16–3.21; *P* = .001), and that of one study conducted in Europe exhibited a significant correlation (HR = 1.11; 95% CI = 1.03–1.20). Nonetheless, 3 studies conducted in America exhibited no distinct correlation (HR = 2.23; 95% CI = 0.96–5.19). The HR of 6 studies that used other methods was 2.29 (95% CI = 1.35–3.91; *P* = .000); nevertheless, 4 articles that used ROC-based threshold method (HR = 1.05;95% CI = 0.98–1.13) did not exhibit any correlation. According to the subgroup analysis stratified by the analysis method, the HR of 4 studies employing multivariate regression analysis was 4.29 (95%CI = 2.67–6.89, *P* = .576), and that of 7 studies employing univariate regression analysis was 1.09 (95%CI = 1.01–1.17; *P* = .002).

A total of 6 studies examined OS with TLG. After combining HRs, we observed that a higher TLG predicted the poorer OS. The random-effects model detected a statistical significance (HR = 1.85; 95% CI = 1.16–2.94; *P* = .000; *I^2^* = 78.3%; Fig. 3D) along with evident heterogeneity among different studies. Additionally, in the present study, a sensitivity analysis was performed to estimate the impact on combined HRs. As a result, the combined HR was not significantly changed after studies were removed sequentially (Fig. S1D, http://links.lww.com/MD/H397), which suggested that our results are reliable. As evidenced by Funnel plots, there was a distinct publication bias. Publication bias was not further explored because of the insufficient number of articles enrolled. In addition, a subgroup analysis was performed upon stratification by analysis, cutoff method, and region (Table 3). According to the subgroup analysis stratified by region, neither 4 studies conducted in Asia (HR = 1.91; 95% CI = 0.91–3.99) nor 2 studies from Europe (HR = 2.02; 95% CI = 0.88–4.66) showed correlations. The HR of 3 studies that used other methods was 1.67 (95%CI = 1.20–2.311; *P* = .230); however, 3 articles using the ROC-based threshold method exhibited no association (HR = 1.88; 95% CI = 0.74–4.79). According to the subgroup analysis stratified by the analysis method, the HR of 5 studies employing multivariate regression analysis was 1.77 (95%CI = 1.09–2.88; *P* = .000), whereas one study employing univariate regression analysis exhibited no correlations (HR = 2.61; 95% CI = 0.84–8.09).

## 4. Discussion

As far as we know, this work is the first to explore the predictive value of MTV and TLG in HNSCC survival. HNSCC, the refractory disease, shows a higher incidence rate globally.^[28]^ As shown in some recent meta-analyses, FDG intake may be used as a predictive marker for survival in various cancers, including head and neck cancer, lung cancer, and breast cancer.^[29–31]^ A total of 15 studies, involving 1292 cases altogether, were included in the present work showed that MTV and TLG were affected by diverse factors. According to this meta-analysis, cases showing a higher MTV had a higher DFS risk and demonstrated lower pooled HRs (HR = 1.34; 95% CI,1.15–1.56; *P* = .000; *I^2^* = 86.0%). Further, MTV and TLG predicted a higher OS risk among cases with low pooled HRs (HR = 1.22; 95% CI, 1.09–1.3; *P* = .000; *I^2^* = 82.4% and HR = 1.85; 95% CI, 1.16–2.94; *P* = .000; *I^2^* = 78.3%, respectively) According to our results, TLG did not significantly predict DFS, which was ascribed to the small sample size (just 5 studies examined DFS with TLG) resulting in insufficient statistical efficiency. Therefore, future studies are warranted to explore whether TLG can predict DFS in patients with HNSCC. Our results should be verified in larger prospective studies.

There was distinct heterogeneity of MTV in the prediction of DFS (*I^2^* = 86.0%; *P* = .000) and OS (*I^2^* = 82.4%; *P* = .000). But certain confounders appear to be possibly affecting the relationship between MTV and survival. For investigating the possible heterogeneity source, subgroup analysis upon stratification by region, analysis, and cutoff method was performed for DFS and OS, with DFS being the endpoint (Table 3). At first, the region was considered to stratify the data into 3 subgroups. A marked decrease in heterogeneity was detected in the Asian group (*I^2^* = 21.3%; *P* = .279), no marked reduction in heterogeneity was observed in the American group, and no significant association was observed in the European group. Secondly, the cutoff method was used to stratify data into 2 subgroups. A marked decrease in heterogeneity was found in the ROC group (*I^2^* = 0.0%; *P* = .988), while those in the other method group exhibited no significant reduction in heterogeneity. Thirdly, groups based on univariate and multivariate analysis for extracting HR were evaluated as a source for the HR heterogeneity. No marked decrease in heterogeneity was detected within the univariate analysis group. Lastly, 4 subgroups classified by survival outcomes were analyzed for HR heterogeneity, among which just the LFS subgroup showed significant heterogeneity reduction (*I^2^* = 0.0%; *P* = .994). Therefore, based on the above results, the region, endpoint, and cutoff method are identified as the heterogeneity sources for DFS. Likewise, region, analysis method, and cutoff method were utilized in the subgroup analysis of OS. Among subgroups based upon univariate and multivariate regression analysis for analyzing HR for OS, the multivariate analysis group exhibited significantly reduced heterogeneity (*I^2^* = 0.0%; *P* = .576). Heterogeneity was not markedly reduced among region-or cutoff method-stratified subgroups. Therefore, based on the above results, the method for regression analysis is identified to be the heterogeneity source of OS.

There was distinct heterogeneity observed for TLG in the prediction of OS (*I^2^* = 78.3%; *P* = .000). As seen with MTV in predicting DFS and OS, there might be certain confounders possibly affecting the relation between the TLG and survival. In order to investigate the source of heterogeneity, data were stratified by the cutoff method into 2 subgroups, and a marked decrease in the heterogeneity was observed in the other methods group (*I^2^* = 32.0%; *P* = .230). However, heterogeneity was not observed to be markedly reduced in the method of regression analysis-and region-stratified subgroups. Based on the above results, the cutoff method was observed to be the heterogeneity source of OS.

Further, TLG and MTV are influenced by SUV (standard uptake value).^[14]^ SUV, in turn, can be affected by some technical or patient-dependent factors, like attenuation correction, fasting time, blood glucose, and uptake time, and therefore, these factors should also be strictly controlled. PET/CT parameters (post-injection interval, duration of fasting, 18 F-FDG doses) in this study were within the normal range^[32–34]^ (Table 3). Although confounders like SUV may affect the association between MTV/TLG and disease prognosis, while the higher MTV and TLG were associated with patient prognosis, the present work did not establish the optimal threshold of TLG or MTV. Therefore, more high-quality studies and methods are required to identify the optimal thresholds for MTV and TLG.

Certain limitations must be noted in this study. Firstly, 13 out of 15 articles that were included in this study were retrospective in nature, and therefore, the robustness of results might be insufficient, thereby inevitably causing biases. Secondly, confounders like SUV could have a certain effect on the survival, TLG, and MTV; but this study did not identify the optimal threshold for TLG or MTV. Thirdly, all the included studies, which mostly had high quality, were evaluated using the Cochrane risk bias tool. Further, several studies did not provide sufficient data related to patients or 18F-FDG PET. Therefore, HNSCC survival data and PET parameters must be investigated further for conclusive analysis. Fourthly, due to the HNSCC heterogeneity, this work enrolled cases of different histological grades, stages, or patients who received different treatments that also could be affecting patient survival and event occurrence. Fifthly, PFS, RFS, EFS, DFS, and LFS were not the same, which might also result in bias. Sixthly, articles published in the English language only were enrolled, leading to the possible language bias. Moreover, study selection and follow-up length are also associated with a high risk of bias and thereby might result in possible inaccuracy. Nevertheless, our results are reliable, as suggested by the assessment of publication bias. However, further multicenter RCTs are needed to validate our findings.

## 5. Conclusion

Although diverse methods were used for HNSCC cases of diverse subtypes, this study confirmed that HNSCC cases with a higher MTV were related to an enhanced risk of side reactions or death, while a high TLG predicted the increased death risk. Based on our findings, TLG did not predict adverse events. Larger studies are warranted for confirming whether PET/CT parameters can be used to predict HNSCC prognosis.

## Acknowledgments

All authors have contributed significantly. All authors are in agreement with the content of the manuscript.

## Author contributions

**Conceptualization:** Aihua Jin.

**Data curation:** Aihua Jin, Yinfeng Cui.

**Formal analysis:** Aihua Jin, Yinfeng Cui.

**Investigation:** Aihua Jin, Xuezhe Yin, Yinfeng Cui.

**Methodology:** Aihua Jin, Xuezhe Yin, Yinfeng Cui, Liguang Ma.

**Project administration:** Aihua Jin, Xuezhe Yin, Yinfeng Cui.

**Resources:** Aihua Jin, Xing Lin, Xuezhe Yin, Yinfeng Cui.

**Software:** Xing Lin, Xuezhe Yin, Yinfeng Cui, Liguang Ma.

**Supervision:** Xing Lin, Xuezhe Yin.

**Validation:** Xing Lin, Liguang Ma.

**Visualization:** Xing Lin, Liguang Ma.

**Writing – original draft:** Xing Lin.

**Writing – review & editing:** Xing Lin.

## Supplementary Material


