# Supplementary figures and images for: Prognostic value of MTV and TLG of 18 F-FDG PET in patients with head and neck squamous cell carcinoma: A meta-analysis
Source: Medicine (Baltimore). 2022 Sep 30;101(39):e30798. doi: 10.1097/MD.0000000000030798 (PMC9524907; doi:10.1097/MD.0000000000030798)

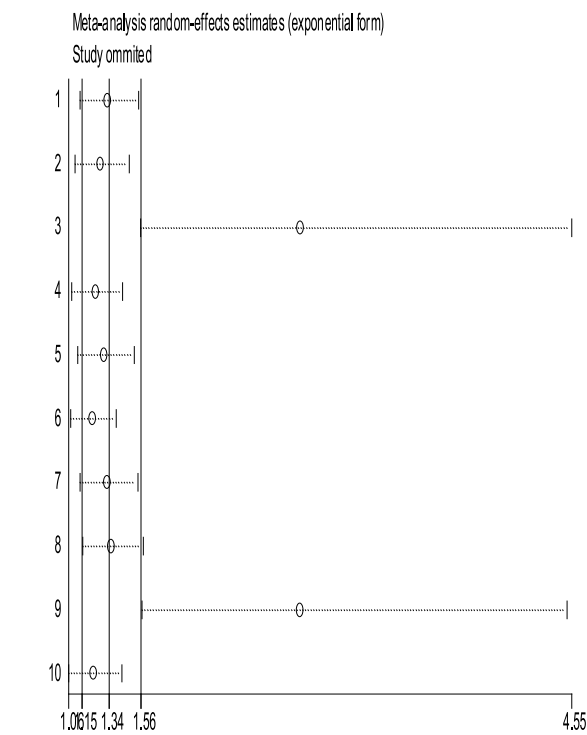

A

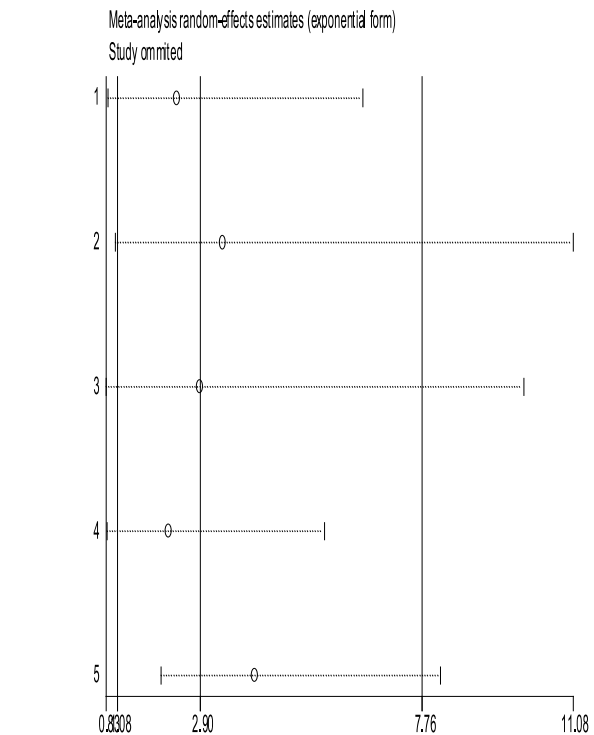

B

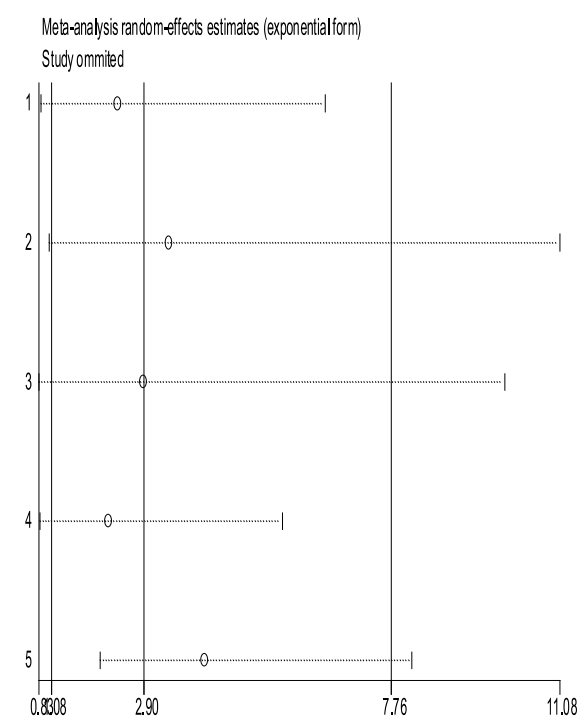

C

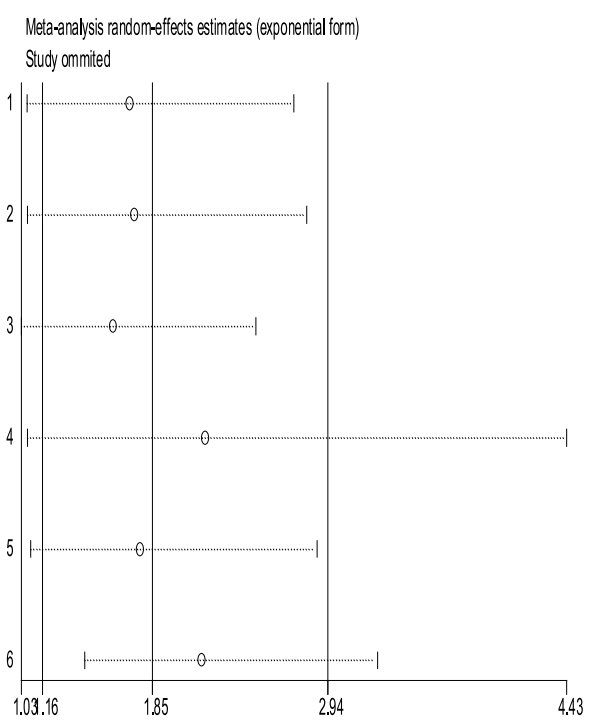

D

Supplement: Supplementary file 1 [file medi-101-e30798-s001.pdf]

A

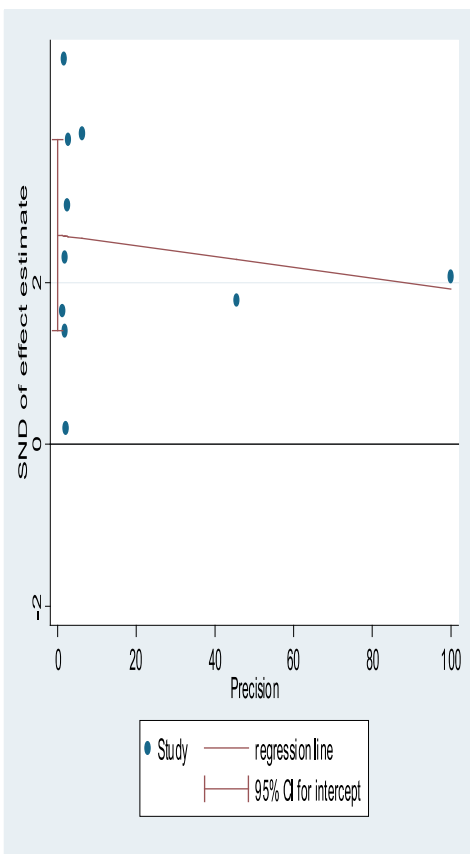

B

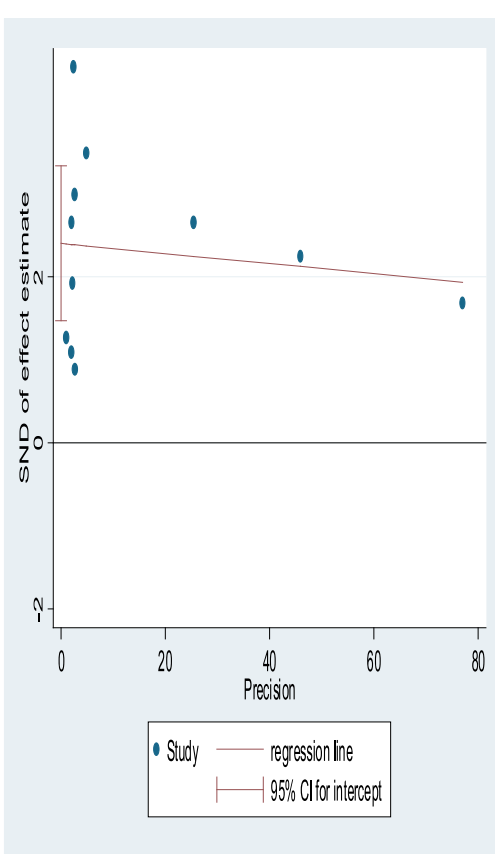

Supplement: Supplementary file 2 [file medi-101-e30798-s002.pdf]
